# Medical Witnesses and Lawyers

**Published:** 1852-06

**Authors:** 


*Medical Witnesses and Lawyers.*—According to the morning Chronicle of Saturday last, whilst the Lord Chancellor was hearing a lunacy case, the previous day, he is reported to have uttered the following observation: "When a medical gentleman, with a large fee, was sent to visit a patient, his report invariably sustained, to a great extent, the view taken by the party employing him."

Disregarding altogether, the animus which apparently dictated such language in a court of justice, we must dissent, *in toto*, from its application to medical practitioners; and would respectfully assert, that the profession are quite as honest in giving their opinion as any barrister, consulted upon a point of law by a solicitor. Lord Truro, having once been an attorney, knows better than we could tell his lordship, that should a counsel decide against a client's right of action, that information is never communicated to the opposite party; nor are adverse witnesses ever called, whose evidence might invalidate the plea brought forward. The same proceeding is always followed respecting medical statements and evidence. If unfavorable to the persons employing the professional gentleman consulted, nothing is said thereon; so that the court is never informed, unless in support of the case then advocated. Therefore, to suppose that medical men always decide in favor of the individual who pays them, is fully as erroneous as it would be for a Lord Chief Justice to observe, during the trial of a case before him, "It is very singular, when counsel with a large fee comes to plead in this court, his oration invariable sustains, to a great extent, the views taken by the party employing him!" How the common law bar would grin at such an effusion, may be easily conceived. Nor would the learned wigs be less astonished, were one of the legal fraternity to quote precedents against his own client, or argue in favor of the opposite one.

Another dictum recently expressed, also, deserves animadversion, when it was said that fifty guineas, given to Dr. Winslow, for visiting a patient in Yorkshire, seemed large. On the contrary, we think the amount was less than half what it ought to have been, considering the time employed, and the responsibility that physician thereby incurred.

Instead of criticising medical practitioners, we would advise all judges to condemn all barristers who take large fees from attorneys, to appear in court when particular cases are called, but can not be found at the critical moment. This would be a much better reform than grudging the honorarium of a medical man, for mental and bodily labor actually performed. With all deference towards the venerable judges of England, we sincerely believe it is injudicious and misplaced, for an occupant of the judicial bench to indulge in tirades which may disparage either his own or any of the other learned professions in public estimation.—*London Lancet, January*, 1852.

